# A randomized trial of an intervention to improve use and adherence to effective coronary heart disease prevention strategies

**DOI:** 10.1186/1472-6963-11-331

**Published:** 2011-12-05

**Authors:** Stacey L Sheridan, Lindy B Draeger, Michael P Pignone, Thomas C Keyserling, Ross J Simpson, Barbara Rimer, Shrikant I Bangdiwala, Jianwen Cai, Ziya Gizlice

**Affiliations:** 1 Division of General Medicine and Clinical Epidemiology, University of North Carolina, Chapel Hill, NC, USA; 2 Center for Health Promotion and Disease Prevention, University of North Carolina, Chapel Hill, NC, USA; 3 Division of Cardiology, University of North Carolina, Chapel Hill, NC, USA; 4 Gillings School of Global Public Health, University of North Carolina, Chapel Hill, NC, USA; 5 Department of Biostatistics, University of North Carolina, Chapel Hill, NC, USA

## Abstract

**Background:**

Efficacious strategies for the primary prevention of coronary heart disease (CHD) are underused, and, when used, have low adherence. Existing efforts to improve use and adherence to these efficacious strategies have been so intensive that they are impractical for clinical practice.

**Methods:**

We conducted a randomized trial of a CHD prevention intervention (including a computerized decision aid and automated tailored adherence messages) at one university general internal medicine practice. After obtaining informed consent and collecting baseline data, we randomized patients (men and women age 40-79 with no prior history of cardiovascular disease) to either the intervention or usual care. We then saw them for two additional study visits over 3 months. For intervention participants, we administered the decision aid at the primary study visit (1 week after baseline visit) and then mailed 3 tailored adherence reminders at 2, 4, and 6 weeks. We assessed our outcomes (including the predicted likelihood of angina, myocardial infarction, and CHD death over 10 years (CHD risk) and self-reported adherence) between groups at 3 month follow-up. Data collection occurred from June 2007 through December 2009. All study procedures were IRB approved.

**Results:**

We randomized 160 eligible patients (81 intervention; 79 control) and followed 96% to study conclusion. Mean predicted CHD risk at baseline was 11.3%. The intervention increased self-reported adherence to chosen risk reducing strategies by 25 percentage points (95% CI 8% to 42%), with the biggest effect for aspirin. It also changed predicted CHD risk by -1.1% (95% CI -0.16% to -2%), with a larger effect in a pre-specified subgroup of high risk patients.

**Conclusion:**

A computerized intervention that involves patients in CHD decision making and supports adherence to effective prevention strategies can improve adherence and reduce predicted CHD risk.

**Clinical trials registration number:**

ClinicalTrials.gov: NCT00494052

## Background

Efficacious strategies for the primary prevention of coronary heart disease (CHD) (including aspirin, blood pressure and cholesterol medicine, and smoking cessation) reduce the relative risk of CHD events by 20-50% each [1-4]. However, these strategies are underused in clinical practice [5-9] and have low rates of adherence [10].

Multiple systematic evidence reviews [11-15] and a recent meta-review [16] have demonstrated that many types of interventions yield small to moderate improvements in medication use and adherence. A common observation, however, is that efficacious interventions are resource-intensive. For instance, the most efficacious interventions for hypertension and cholesterol medication adherence include multiple telephone or in-person counseling sessions delivered by researchers, nurses, or pharmacists with or without the addition of other approaches, such as medication reminders [13,17]. This led one recent systematic review to conclude that "[current adherence trials] provide little evidence that medication adherence can be improved consistently within the resources available in clinical practice." [13]

Given limited resources for counseling in clinical practice [18], alternatives to medication use and adherence counseling must be considered and tested. Two such options are decision aids and automated message libraries. Decision aids employ interactive media to convey health information, help patients clarify their values, and choose health options that are consistent with their values. Automated message libraries generate individually tailored messages that can be used to address specific patient barriers, provide skill building, and give information about ancillary resources [19]. Both have the potential to alleviate provider burden for counseling. However, their role in promoting medication initiation and adherence is largely untested [13,20-22].

This paper reports on the effects of an intervention designed to promote initiation and adherence to efficacious strategies for the primary prevention of CHD. The intervention includes both a decision aid and a series of automated tailored adherence reminders.

## Methods

### Overview

**Figure 1:**
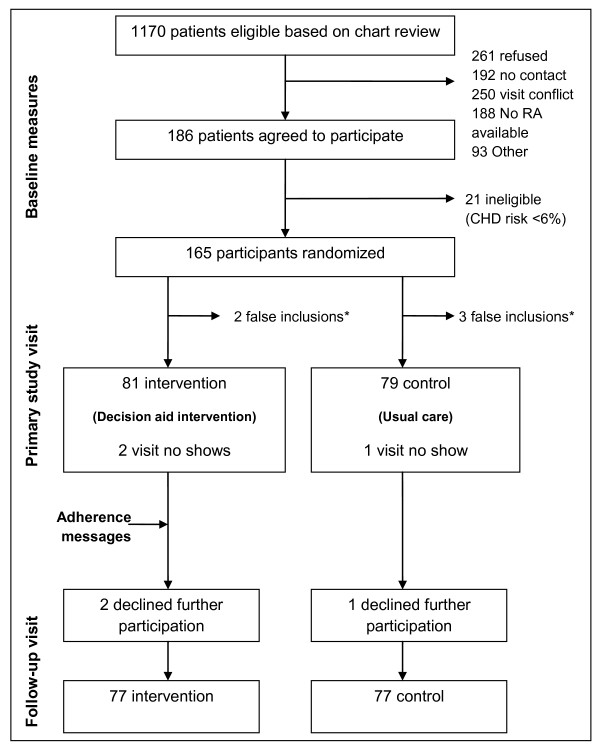
**Study Flow Diagram**. Abbreviations: RA = Research Assistant; CHD = Coronary Heart Disease. * Ineligible after measuring baseline risk factors (CHD risk < 6%).

To test the feasibility of delivering the intervention in clinical practice and the effect of the intervention on important efficacy outcomes, the research team conducted a randomized trial at one university general internal medicine practice between June 2007 and December 2009. After collecting baseline data, study staff randomized participants to either the intervention or usual care, and then saw them for two additional study visits over 3 months. (See Figure 1) The University of North Carolina at Chapel Hill's Biomedical Institutional Review Board (IRB) approved and monitored this study.

### Setting

The research team conducted this trial in one university internal medicine practice staffed by 94 physicians, including 18 attendings and 76 residents who were not part of the study team and were eligible for participation. 94% of eligible attendings and 32% of eligible residents attended a study education session, agreed to participate, and allowed their patients to be enrolled. The 1-hour education session encouraged physicians to 1) engage their patients in decisions about CHD prevention and 2) use readily available resources to support adherence. Prior to the education session, physicians gave written informed consent.

### Patient Population

Patients were eligible to participate if they were presenting for routine care with an enrolled physician; were between the ages of 40-79 years; had no prior history of cardiovascular disease, diabetes mellitus, or other serious medical condition that limited their life expectancy to less than 5 years; and were at moderate (6-10%) or high risk (> 10%) of CHD over 10 years based on a Framingham risk equation [23]. Patients were excluded if they were presenting for their first visit, had no cholesterol check within the past 18 months, were unable to speak or read English, or had extreme elevations of systolic blood pressure (> 180 mmHg) or cholesterol (> 300 mg/dL).

### Patient Recruitment and Enrollment

To recruit patients, study staff identified all age eligible patients returning for visits with enrolled physicians. After reviewing patients' charts, staff mailed recruitment letters to potentially eligible patients and made 3 attempts to call patients who did not "opt-out" of further contact by returning an opt-out postcard. When they reached patients by phone, staff reassessed eligibility and arranged a visit for interested patients.

Patients presented for their initial study visit at least one week prior to a regularly scheduled clinic visit with an enrolled physician. During this visit, patients provided written informed consent; filled out a baseline survey about their demographics, medication use, and plans and self-efficacy for risk reduction; and had their CHD risk factors assessed. Study staff used baseline risk factor assessments to recalculate baseline predicted CHD risk and assure study eligibility.

### Randomization and Blinding

Following enrollment, patients were randomized by study staff who accessed an online randomized schedule. Staff told patients only that they were participating in a study about "prevention of heart disease." Physicians were not blinded and saw patients in both the intervention and control group.

### Patient-Directed Intervention

The patient-directed intervention consisted of two components: a computerized decision aid to promote initiation of effective CHD prevention strategies, and a series of automated mailed tailored messages to promote adherence. Intervention components were based on the Integrative Theory, Protection Motivation Theory, and Self-Determination Theory as previously described [24]. Further, they were designed at a 6-8th grade reading level and pre-tested using cognitive and usability testing.

#### Decision Aid

The web-based decision aid, called Heart to Heart, had five main functions: it 1) calculated patients' overall risk of CHD events in the next 10 years using a continuous Framingham equation [23]; 2) educated patients about CHD, their predicted global CHD risk, their risk factors, and the benefits and harms of the most effective risk-reducing strategies (aspirin, cholesterol medication, hypertension medication, and smoking cessation); 3) helped patients clarify their values [25]; 4) encouraged them to choose risk-reducing strategies that would be acceptable and feasible to them for long-term CHD risk reduction; and 5) coached them to communicate their decisions with their physicians by providing audio clips about ways to overcome common communication barriers. Investigators pilot-tested a previous version of the decision aid [26] and studied its effects with and without the values clarification section [25]. The coaching portion has not been independently described or tested; a more detailed description is provided in Additional File 1.

#### Tailored Adherence Messages

**Figure 2:**
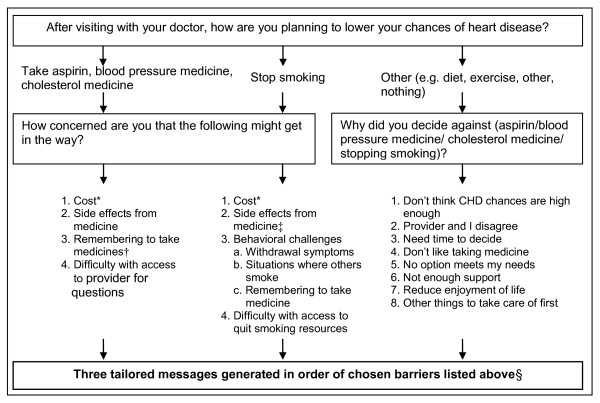
**Basic Logic and Structure of Tailored Messages**. Abbreviations: CHD = Coronary Heart Disease. * Further tailored on whether participant has prescription drug plan. † Further tailored on self-reported adherence at baseline. ‡ Further tailored on which smoking cessation medication was prescribed. § If fewer than 3 barriers identified, patients received default messages: "Plan Ahead"; "Check on Your Progress Regularly"; and "Eat Right and Be Active".

The tailored messaging system helped patients circumvent self-identified barriers and gain the necessary resources and skills for adherence. This automated messaging system included a library of 76 unique messages, which could be combined in over a million combinations in response to a few brief survey questions. The basic logic of this tailoring is shown in Figure 2; more detailed content is provided in Additional File 2. In brief, messages were first tailored based on patients' intentions to start one or more of the most effective risk reducing strategies (e.g. take aspirin, cholesterol medication, hypertension medication, or stop smoking). For patients who planned to start risk reducing strategies, messages then addressed self-identified barriers to adherence, including cost; side effects; difficulty remembering to take medications; difficulty accessing care; and, for smokers, behavioral challenges in stopping smoking. For patients who did not plan to start risk reducing strategies, messages encouraged reconsideration of the need for global CHD risk reduction and addressed self-identified barriers to risk reduction. Based on the logic in Figure 2, each participant in the intervention group received a series of 3 computer-generated mailed tailored adherence newsletters.

### Delivery of the Intervention and Surveys

The primary study visit occurred in conjunction with a previously scheduled visit with a participant's usual physician. Participants randomized to the intervention group presented 45 minutes early to their clinic visit to view the decision aid and received a list of their risk factors to get them started. They then self-entered their risk factors and navigated the decision aid on their own using a computer stationed in a private room in the clinic. They had access to research staff at all times for questions about navigation. After viewing the decision aid, participants proceeded to their clinic visit. Participants randomized to the control group did not receive a decision aid and proceeded directly to their clinic visit. Following the clinic visit, a research assistant administered surveys to both intervention and control patients to assess their plans for CHD risk reduction. She then mailed tailored adherence messages to participants in the intervention group approximately 2 weeks, 4 weeks, and 6 weeks after their initial study visit.

Approximately 3 months after the initial study visit, all participants presented for a final study visit and final measures.

### Measures

#### Feasibility of Intervention Delivery

Investigators measured the feasibility of intervention delivery by measuring the total time participants spent with the decision aid, whether or not participants required assistance with the decision aid, and the time spent by the research assistant preparing and mailing tailored messages.

#### Change in Predicted Global CHD Risk

Investigators measured predicted global CHD risk (i.e. risk of angina, myocardial infarction (MI), and CHD death over 10 years) using a well-validated Framingham risk equation [23], that combined information about a patients' age, gender, smoking history, diabetes status, systolic blood pressure level, total and HDL-cholesterol levels, and left ventricular hypertrophy status in a multivariate equation. Following a precedent set by other researchers [27-30], the research team assumed that the benefits of medication are reasonably captured by entering revised risk factor data into the Framingham equation. Because the effect of aspirin use on CHD risk is not accounted for by the Framingham equation, the team additionally performed a priori-specified modeling of the effects of aspirin by applying a 28% risk reduction [1] to the calculated CHD risk. This modeling mirrored the modeling of aspirin effects participants saw in the decision aid [31]. To be consistent with a more recent meta-analysis published during the study period [32], the team also performed a sensitivity analysis, recalculating predicted global CHD risk using a 23% risk reduction for men and a 0% reduction for women. The team also performed sensitivity analyses to determine whether modeling the effects of each CHD risk reducing strategy (aspirin, blood pressure medicine, cholesterol medicine, and smoking cessation) on baseline CHD risk produced different results than recalculating risk as described above. For this analysis, the team multiplied baseline Framingham risk scores by a 28% relative risk reduction for aspirin users, a 25% relative risk reduction for blood pressure medicine users, a 30% relative risk reduction for cholesterol users, and a 50% relative risk reduction for those who stopped smoking [1-4]. Modeling medicine effects may allow investigators to estimate the full benefits of medicines (including those which accrue outside changes in traditional CHD risk factors), however, modeling doesn't adequately account for variance in medicine adherence or the effects of other co-interventions (such as diet and exercise) that impact CHD risk.

#### Intent to Start Any Effective CHD Risk Reduction Strategy

The research team measured intent to start any effective risk reducing strategy after the primary study visit by asking participants whether or not they planned to initiate aspirin or smoking cessation or initiate or increase blood pressure or cholesterol medication.

#### Self-reported Adherence

Adherence was assessed at 3 months both by self-reported adherence to any new or escalated risk reduction strategy and by self-reported adherence to each individual strategy separately. To verify self-reported adherence, the team then examined changes in CHD biomarkers (including blood pressure and total and HDL cholesterol) among those who self-reported adherence.

#### CHD Risk Factors

Study staff measured CHD risk factors at baseline and follow-up study visits using well-defined protocols. They measured blood pressure using a non-invasive oscillometric automatic monitor (Omron HEM-907) after the patient had been seated for at least 5 minutes. They averaged three measurements taken at one minute intervals, and defined hypertension as a systolic measurement > 140 mm Hg [33]. They measured serum total and HDL cholesterol levels at the UNC Hospitals' McLendon Laboratory using enzymatic calorimetric testing (Roche Diagnostics Corporation) and defined high cholesterol as a total/HDL cholesterol ratio > 4 [34]. Finally, staff measured smoking status by self-report with confirmation by urine dipstick using the NicAlert test strips (Jant Pharmacal; Encino, CA). A test strip measure of 3 or higher (cotinine concentration > 100 ng/mL) is a positive indication of tobacco use.

### Statistical Considerations

#### Sample Size

Sample size was not based on hypothesis testing, but instead on a reasonable estimation of the sample size necessary to 1) assess the feasibility of the intervention, and 2) determine the effect sizes for the main effects of the intervention.

#### Analysis Methods

All data analysis was performed using SAS (Cary, NC) software. To examine the effects of the intervention on primary and secondary efficacy outcomes, including predicted CHD risk, intent to start therapy, self-reported adherence to therapy, and changes in risk factors, investigators used mixed effects models. The study team used a logistic link function for binary outcome variables. They used linear mixed effects models for the continuous outcome variables. Each model included the intervention, education as fixed effects and physicians as random effects. Models on CHD risk factors additionally included baseline measures of the pertinent CHD risk factors as fixed effects. As a check on self-reported adherence, analyses examined the effect of self-reported adherence on CHD risk factors among those who self-reported adherence to blood pressure and cholesterol medication.

To test intent for CHD risk reduction and self-reported adherence as mediators of the intervention's effect on predicted CHD risk, the team used generalized linear mixed effects models and the Baron and Kenny 4-step approach to mediation analysis [35].

## Results

The study sample included 24 eligible physician participants who had patients enrolled in the study and 160 patient participants who agreed to participate and were randomized (see Table 1), 81 in the intervention group and 79 in the control group. The study lost 6 patient participants during follow-up, resulting in a 96% follow-up rate (see Figure 1).

**Table 1:** Baseline Participant Characteristics

Characteristic	Total Group (N = 160*)	Intervention Group (N = 81)	Control Group (N = 79)
Mean age	63	63	64
Female	28%	27%	28%
Race:			
White	86%	88%	84%
Black	10%	10%	10%
Education:			
At least some college	90%	98%	82%†
Enrolled in a prescription drug plan	90%	91%	89%
Missed medicine in the last month:			
Less than 5% of time	92%	90%	95%
6-25% of time	4%	4%	3%
26-50% of time	2%	3%	2%
51-75% of time	0%	0%	0%
76-95% of time	1%	1%	0%
More than 95% of time	1%	1%	0%
Have potentially modifiable CHD risk factors:			
Blood pressure > 140/90 (mmHg)	36%	35%	37%
Total cholesterol/HDL ratio > 4	54%	53%	51%
Smoker	13%	14%	13%
Not using aspirin, but eligible for it	50%	54%	47%
Mean systolic blood pressure (mmHg)	136.9	136.2	137.6
Mean diastolic blood pressure(mmHg)	81.0	81.5	80.4
Mean total cholesterol (mg/dL)	201.1	204.9	197.1
Mean HDL cholesterol (mg/dL)	53	54.1	51.9
Mean predicted CHD risk over 10 yrs	11.3	11.2	11.4
# of possible intervention options for CHD risk:‡			
0	10%	12%	8%
1	28%	28%	27%
2	47%	40%	54%
3	16%	20%	11%
4	0%	0%	0%
Have self-efficacy to lower at least 1 CHD risk factor	98%	99%	96%
Are planning best evidence interventions§	27%	28%	25%

Abbreviations: CHD = Coronary heart disease; HDL = high density lipoprotein

* 160 participants at baseline; 3 missed both primary and follow-up study visits and 3 declined further participation after the primary study visit

† Statistically different between the intervention and control groups (p < .01)

‡ This includes hypertension medicine, cholesterol medicine, smoking cessation, and aspirin

§Proportion planning to do any of the following intervention options that are supported by the highest quality of evidence: hypertension medicine, cholesterol medicine, smoking cessation, and aspirin

### Feasibility of Intervention Delivery in Practice

Intervention participants spent an average of 12 minutes (range: 1-45 minutes) with the decision aid and were able to navigate it with minimal assistance. Tailored message mailings took research assistants less than 5 minutes per participant.

### Effect of the Intervention on Predicted Global CHD Risk

At follow-up, participants in the intervention group had a statistically significant lower mean 10-year predicted CHD risk than participants in the control group (adjusted absolute difference -1.1%; 95% CI -2.0% to -.16%). In a pre-specified high risk subgroup, the effect was larger, but not statistically significant due to the smaller sample (see Table 2). In sensitivity analyses assuming a 23% relative risk reduction for aspirin for men and a 0% relative risk reduction for aspirin for women, results were similar in magnitude (adjusted absolute difference, entire sample: -1.0%; 95% CI -2.0% to 0.02%; high risk subgroup: -1.53%; 95% CI -3.47% to 0.42%; moderate risk subgroup: -0.95%; 95% CI -2.33% to 0.44%), but not statistically significant. In sensitivity analyses that modeled the effect of risk-reducing strategies (rather than recalculating risk), results were also similar in magnitude (adjusted absolute difference, entire sample: -1.0%, 95% CI -2.4% to 0.30%; high risk subgroup: -1.5%, 95% CI -3.7% to 0.70%; moderate risk subgroup: -0.85%. 95% CI -1.5% to -0.16%), although again not statistically significant.

**Table 2:** Effect of Heart to Heart Intervention on CHD Risk at Follow-up

	Control group (N = 77)	Intervention group (N = 77)	Absolute difference (95% CI) *	**Adjusted absolute difference**†
Total group (n = 154)	10.4%	9.1%	-1.3% (-3.0% to 0.40%)	-1.1% (-2.0% to -0.16%)
Moderate risk at baseline (6-10%) (n = 82)	7.9%	7.0%	-0.93% (-2.4% to 0.51%)	-0.75% (-1.6% to 0.13%)
High risk at baseline (> 10%) (n = 72)	13.2%	11.4%	-1.72% (-3.83% to 0.38%)	-1.4% (-3.2% to 0.39%)

Abbreviations: CHD **= **Coronary heart disease

*Adjusted for random effects of clustering within physician

†Adjusted for baseline CHD risk, education level, and random effects of clustering within physician

### Effect of the Intervention on Intent to Start Any Effective CHD Risk Reducing Strategy

Compared with the control group, participants in the intervention group had significantly higher intentions to start or increase any of the effective CHD risk reducing therapies promoted by our intervention (control 42%, intervention 63%; absolute difference 21%; 95% CI 5% to 37%; adjusted p < 0.01). Increases in overall intent primarily resulted from increases in intent to take aspirin (control 24%, intervention 43%; absolute difference 19%; 95% CI -1 to 39%) and cholesterol medication among those with abnormal cholesterol (control 9%, intervention 39%; absolute difference 30%; 95% CI 14 to 46%).

### Effect of the Intervention on Self-reported Adherence to Chosen Risk Reducing Therapies

Patients in the intervention group also had higher self-reported adherence to the chosen risk reducing therapies promoted by the intervention (adjusted absolute difference +25%; p < 0.01; see Table 3). Most of the interventions' effect on adherence seemed to be through aspirin use (+36%; 95% CI 17% to 55%; p < 0.01), with little effect on blood pressure or cholesterol medication, however, the sample sizes for these latter estimates were small and underpowered.

**Table 3:** Proportion of Participants Adhering to their Chosen CHD Risk Reduction Therapy at 3-month Follow-up

Chosen therapy (number in analysis)	Control % (n/N)	Intervention %(n/N)	Absolute difference (95% CI) (p-value)*	Odds ratio (95% CI)*	Adjusted absolute difference, p-value†	Adjusted odds ratio (95% CI) †
Any chosen therapy promoted by intervention (n = 149) ‡	34% (25/73)	59% (45/76)	25% (8% to 42%) (p < 0.01)	2.8 (1.3 to 5.9)	25%, p < 0.01	3.4 (1.4 to 6.9)
Any chosen therapy, including other§ (n = 154)	68% (52/77)	83% (64/77)	16% (4% to 28%) (p = 0.02)	2.4 (1.2 to 4.9)	14%, p = 0.04	2.2 (1.0 to 4.8)
Take aspirin (n = 51)	58% (11/19)	94% (30/32)	36% (17% to 55%) (p < 0.01)	10.9 (2.0 to 59)	39%, p < 0.01	12.8 (2.0 to 84)
Take cholesterol medicine (n = 20)	83% (5/6)	86% (12/14)	3% (-28% to 33%)	1.2 (0.1 to 12.3)	--	--
Take blood pressure medicine (n = 21)	92% (11/12)	100% (9/9)	8% (-9% to 25%)	--||	--	--
Stop smoking (n = 13)	20% (1)	25% (2)	--	--	--	--

Abbreviations: CHD = Coronary Heart Disease

* Adjusted for clustering within physician

† Adjusted for education level, and random effects of clustering within physician

‡ "Any chosen therapy promoted by intervention" includes hypertension med, cholesterol med, smoking cessation, aspirin

**§ **"Any chosen therapy, including other" includes hypertension med, cholesterol med, smoking cessation, aspirin, diet, and physical activity

|| Unable to calculate due to small cell size

### Effect of the Intervention on CHD Risk Factors

In subgroup analyses, the effect of the intervention on CHD risk factors varied (see Table 4). The intervention tended to reduce blood pressure (adjusted absolute difference, systolic blood pressure: -6.6 mm Hg; 95% CI -14.3 to 1.2; diastolic blood pressure: -1.2 mmHg; 95% CI -5.2 to 2.8), although results didn't achieve statistical significance. It, however, had no effect on total or HDL cholesterol or smoking, although subgroups were small and underpowered.

**Table 4:** Effect of Heart to Heart Intervention on CHD Risk Factors at 3-month Follow-up*

Risk factor (number with risk factor at baseline)	Control group Mean (n or n/N)	Intervention group Mean (n or n/N)	Absolute difference (95% CI) †	**Adjusted absolute difference (95% CI) **‡
Systolic blood pressure, if HTN (n = 53)	146.6 (27)	139.3 (26)	-7.21 (-14.4 to -0.03)	-6.6 (-14.3 to 1.2)
Diastolic blood pressure, if HTN (n = 53)	80.2 (27)	80.4 (26)	+.27 (-4.24 to 4.79)	-1.2 (-5.2 to 2.80)
Total cholesterol, if abnormal cholesterol (n = 67)	196 (33)	203 (34)	+7.02 (-14.8 to 28.8)	+8.0 (-12.0 to 28.1)
HDL, if abnormal cholesterol (n = 67)	42 (33)	46 (34)	+4.3 (0.08 to 8.53)	+1.1 (-3.08 to 3.37)
% Smoking, if smokers (n = 13) §	100% (5/5)	88% (7/8)	--	

Abbreviations: HTN = hypertension; HDL = high density lipoprotein

* Among individuals with baseline risk factors

**† **Confidence intervals adjusted for random effects of clustering within physician

‡Confidence intervals adjusted for education, baseline risk factor levels, and random effects of clustering within physician

§ Confirmed by urinary cotinine

Analyses to confirm self-reported adherence did confirm an effect of self-reported adherence on systolic blood pressure (-8.6 mmHg, 95% CI -14.8 to -2.3) and total cholesterol (-45.6 mg/dL, 95% CI -75.2 to -16.1) in individuals self-reporting adherence to blood pressure and cholesterol medications respectively.

### Potential Mediators of Intervention Effect

In mediation analysis, adding intentions for CHD risk reduction to the model testing the relationship between the intervention and predicted CHD risk reduced the difference in predicted CHD risk by 0.18 absolute percentage points, while adding self-reported adherence to the model reduced the difference in predicted CHD risk by 0.22 absolute percentage points.

## Discussion

At 3-month follow-up, a computerized intervention designed to promote CHD prevention medication use and adherence increased self-reported adherence by 25 percentage points and reduced 10-year predicted global CHD risk by 1.1 percentage points, with a larger effect seen in a pre-specified high risk sub-group. The effect on predicted CHD risk was mediated, in part, by intent to reduce CHD risk and by self-reported adherence.

These findings are similar to those reported in previous studies of more resource-intensive interventions for CHD prevention. For instance, multiple systematic reviews have shown 10-25 absolute percentage point increases in adherence, with the largest effects in studies using multiple telephone or in-person counseling sessions [17]. Furthermore, a recent systematic evidence review found that global risk information, in repeated doses or with repeated counseling, reduced predicted CHD risk by 0.2 to 2 absolute percentage points [36]. What is noteworthy about this study is that it achieved its effects using a computerized decision aid and automated message library to perform the functions of education and counseling.

Also noteworthy about this study are the reported effects of the intervention on aspirin adherence. Given the risk based recommendations for aspirin therapy [37] and individuals' desire for inexpensive, familiar, trusted interventions [24], it is not surprising that the intervention had its greatest effect on initiation of and adherence to aspirin.

What is somewhat surprising about this study is the apparent lack of effect of our intervention on cholesterol-lowering medication adherence. Not only are cholesterol guidelines risk-based, but a recent systematic review [36] found consistent reductions in total and LDL cholesterol in patients who received predicted CHD risk information and counseling. We suspect the lack of effect of the intervention on cholesterol is due in large part to the small number of participants (n = 20) choosing, initiating and adhering to cholesterol therapy, which limits the ability to detect any effect.

To better understand the effects of our intervention, investigators performed mediation analyses. These analyses suggest that both intent to reduce CHD risk and self-reported adherence to medications partially mediate reductions in 10-year predicted CHD risk. The proportional effects, however, are small (~20%), raising questions about other potential mediators such as diet and physical activity.

Interpretation of these findings should proceed with acknowledgement of the following limitations. First, this was a relatively small study not designed to look at subgroup effects. Subgroup analyses are small and underpowered, and should be used only for hypothesis generation. Second, physicians saw patients in both the intervention and control groups, which may have resulted in contamination between study groups. This may have reduced the effects of our intervention. Third, the effect of the intervention was diluted by the presence of participants (10%) who had no options for CHD risk reduction. Fourth, investigators measured adherence primarily by self-report (although with correlation with biomarkers). Based on studies comparing objective and subjective measures of adherence [38], it is likely we slightly overestimated adherence. Fifth, although randomization is expected to result in equally distributed characteristics between the intervention and control groups, the difference in education between groups raises questions about unmeasured confounders. Sixth, the study team conducted this study in one academic medicine clinic, and results might not generalize to other clinics, physicians, or patient populations. Finally, this was a short-term study with only 3 months of follow-up; longer follow-up likely would affect results.

## Conclusions

Limitations aside, the results of this study preliminarily suggest that an adherence intervention employing a computerized decision aid and automated adherence messages can increase patients' adherence and reduce CHD risk. This result should be confirmed in larger and broader populations with longer term follow-up. Additionally, future studies should consider additional implementation issues, such as cost, cost-effectiveness, and alternate mechanisms of delivery (e.g. via electronic health record).

## Competing interests

The authors declare that they have no competing interests.

## Authors' contributions

SS conceived of the study and its design, participated in data analysis, and wrote the manuscript. LD participated in acquisition of data, data analysis, and manuscript drafting. MP, TK, RS, and BR participated in study design and reviewed the manuscript critically for intellectual content. SB participated in study design and data analysis. JC participated in data analysis. ZG participated in study design and performed data analysis. All authors read and approved the final manuscript.

## Pre-publication history

The pre-publication history for this paper can be accessed here:

http://www.biomedcentral.com/1472-6963/11/331/prepub

## Supplementary Material

Additional file 1 **Coaching Portion of the Heart to Heart Decision Aid**. Click here for file

Additional file 2 **Content of Tailored Adherence Messages**. Click here for file
