# Supplementary material for: A randomized trial of an intervention to improve use and adherence to effective coronary heart disease prevention strategies
Source: BMC Health Serv Res. 2011 Dec 5;11:331. doi: 10.1186/1472-6963-11-331 (PMC3268742; doi:10.1186/1472-6963-11-331)
Supplement: Additional file 1 — Coaching Portion of the Heart to Heart Decision Aid. [file 1472-6963-11-331-S1.DOC]

**Additional File 1. Coaching Portion of the Heart to Heart Decision Aid.**

The coaching tool we embedded within our Heart to Heart decision aid 1) outlines the benefits of participating with the provider in decision making about CHD risk reduction (e.g. learning about special issues unique to one’s health, getting information about resources that may help them accomplish their plan), 2) provides a menu of seven common barriers people have in talking with their doctor about their plans (e.g. the doctor decides the agenda, the doctor uses too much medical talk, the doctor doesn’t acknowledge previous successes – see Figure below), 3) provides audio clips matched with still photos of a narrator and patients talking about simple practical ways to overcome common barriers, and 4) produces a summary sheet of the

patient’s experience with Heart to Heart that can be taken to one’s provider to initiate discussion. To increase relevance and engagement for individuals who are diverse in age and racial composition, the coaching tool includes photographs and voices representing a variety of demographics.
