# Supplementary material for: A randomized trial of an intervention to improve use and adherence to effective coronary heart disease prevention strategies
Source: BMC Health Serv Res. 2011 Dec 5;11:331. doi: 10.1186/1472-6963-11-331 (PMC3268742; doi:10.1186/1472-6963-11-331)
Supplement: Additional file 2 — Content of Tailored Adherence Messages. [file 1472-6963-11-331-S2.DOCX]

**Additional File 2. Content of Tailored Adherence Messages**

| **Barrier to Medication Adherence*** | **Specific Content of Tailored Message†‡** |
| --- | --- |
| Cost | -Ask for generic medication  -If you have insurance, ask for medications on the formulary  -Buy medication at price clubs/Walmart  -If necessary, sign up for medication assistance programs  (options and contact info provided) |
| Side effects | - Most people don’t feel different on medications  -If you think you are having side effects, contact your doctor rather than stopping medication on your own  -Many side effects go away as your body gets used to them  -If you have side effects with one medication, you may tolerate other medications just fine  -Engage in appropriate monitoring and follow instructions to reduce the likelihood of side effects |
| Remembering to take medication | -Develop a special routine for taking medicines  -Consider reminders, including pillboxes, medication charts, medication calendars, watches with alarms (resources provided)  -Plan ahead if you won’t be at home when your medication is due |
| Access to resources (incl. provider, pharmacist, smoking quitlines) | -Have ready contact information for your doctor for questions/medication refills (contact info provided)  -Rely on your pharmacist to place refill requests with your doctor and answer questions  -Schedule an appointment if you want to talk more about medications (contact info provided)  -If you are a smoker, call a smoking quitline or try an online program for extra support (contact info and details provided)  -Plan ahead when dealing with others to avoid lapses in medication |
| Behavioral challenges associated with quitting smoking | -Use medications to avoid withdrawal symptoms  -Make lifestyle changes to avoid stress and weight gain  -Enlist support from family, friends, doctor, support groups, online counseling programs  -Avoid situations where others will be smoking |

*Provided in order of importance to individual

**†** Tailored to the specific medication chosen

**‡** Participants who did not indicate barriers to taking medication received 3 default messages: Plan Ahead to Take Your Medicine; Check Your Progress Regularly; and Eat Right and Be Active
